# Emergence of Ceftriaxone-Resistant *Salmonella* Isolates and Rapid Spread of Plasmid-Encoded CMY-2–Like Cephalosporinase, Taiwan

**DOI:** 10.3201/eid0903.010410

**Published:** 2003-03

**Authors:** Jing-Jou Yan, Wen-Chien Ko, Cheng-Hsun Chiu, Shu-Huei Tsai, Hsiu-Mei Wu, Jiunn-Jong Wu

**Affiliations:** * National Cheng Kung University, Tainan, Taiwan; † Chang Gung Children’s Hospital, Taoyuan, Taiwan

**Keywords:** *Salmonella*, *Escherichia coli*, *Klebsiella pneumoniae*, cephalosporin, cephalosporinase, research

## Abstract

Of 384 *Salmonella* isolates collected from 1997 to 2000 in a university hospital in Taiwan, six ceftriaxone-resistant isolates of *Salmonella enterica* serovar Typhimurium were found in two patients in 2000. The resistance determinants were on conjugative plasmids that encoded a CMY-2–like cephalosporinase. During the study period, the proportion of CMY-2–like enzyme producers among *Escherichia coli* increased rapidly from 0.2% in early 1999 to >4.0% in late 2000. *Klebsiella pneumoniae* isolates producing a CMY-2–like β-lactamase did not emerge until 2000. The presence of *bla*_CMY_-containing plasmids with an identical restriction pattern from *Salmonella*, *E. coli*, and *K. pneumoniae* isolates was found, which suggests interspecies spread and horizontal transfer of the resistance determinant. Various nosocomial and community-acquired infections were associated with the CMY-2–like enzyme producers. Our study suggests that the spread of plasmid-mediated CMY-2–like β-lactamases is an emerging threat to hospitalized patients and the public in Taiwan.

Because of increasing rates of antimicrobial resistance in salmonellae worldwide (*1*–*4*), extended-spectrum cephalosporins, especially ceftriaxone, are frequently used to treat invasive salmonellosis. Since the early 1990s, ceftriaxone-resistant salmonellae have been noted in many countries, including France, Argentina, Algeria, Tunisia, Turkey, Spain, Latvia, the United States, and Hungary (*5*–*17*), with resistance conferred by various class A extended-spectrum β-lactamases or class C cephalosporinases (*18*). These β-lactamases in salmonellae are usually encoded on transmissible plasmids (*5*–*11*,*13*–*17*), which could be acquired from other multidrug-resistant Enterobacteriaceae, such as *Klebsiella pneumoniae* or *Escherichia coli* (*10*,*19*).

Although the prevalence of multidrug-resistant salmonellae is a major public health concern, ceftriaxone-resistant salmonellae have not yet been reported in Taiwan (*4*,*20*,*21*). We conducted a retrospective survey of clinical *Salmonella* isolates collected over a 4-year period in a teaching hospital to investigate whether ceftriaxone-resistant salmonellae have emerged in Taiwan. Two ceftriaxone-resistant strains producing a CMY-2–like class C cephalosporinase were found. We also established a connection between the appearance of the resistant strains and the rapid spread of the *bla*_CMY-2_-like gene in this area.

## Materials and Methods

### Bacterial Isolates and Patients

From January 1997 to December 2000, a total of 384 *Salmonella* isolates from 324 patients were collected at the National Cheng Kung University Hospital, a tertiary-care referral center with 900 beds in southern Taiwan. According to the criteria of the National Committee for Clinical Laboratory Standards (NCCLS) for the disk diffusion method, we selected for further investigation the isolates that exhibited resistance or intermediate resistance to cefpodoxime, ceftazidime, aztreonam, cefotaxime, ceftriaxone, cefoperazone, or cefixime (*22*). For comparison, 5,520 *E. coli* isolates and 3,680 *K. pneumoniae* isolates collected during the same period were included. Of these isolates, 1,210 nonrepetitive *E. coli* isolates collected from January to September 1999 were investigated previously (*23*). The 1997 and 1998 isolates were randomly collected and the 1999 and 2000 isolates were consecutively collected. *Salmonella* isolates were serotyped according to the Kauffman and White scheme (*24*) by using somatic and flagellar antigens (Becton Dickinson Microbiology, Cockeysville, MD).

We reviewed the medical records of patients infected with or colonized by the organisms being studied. Patients who provided samples positive for the organisms collected from any body site but who had no related signs or symptoms of infections were considered colonized. Nosocomial acquisition of infections in the teaching hospital was defined according to the 1988 definitions from the Centers for Disease Control (*25*). Patients transferred from other hospitals or nursing homes with infections occurring <48 h after admission were considered to have acquired the infections at the other locations. Hospitalization histories for the previous locations were recorded for outpatients and for inpatients who were colonized by the studied organisms and who provided samples within 48 h after admission.

### Susceptibility Testing

The susceptibilities of isolates to antimicrobial agents were determined by using the agar dilution or disk diffusion method according to the NCCLS guidelines (*22*,*26*). The antimicrobial agents used for the agar dilution test included amoxicillin, clavulanic acid, ceftriaxone, cefoxitin, ceftazidime, cefotaxime, and imipenem. Sources of antimicrobial agents used in this study are described elsewhere (*23*). Breakpoints used for susceptibility meet NCCLS criteria; the ceftazidime, cefotaxime, and ceftriaxone breakpoint was 8 µg/mL (*26*).

### Isoelectric Focusing

Crude β-lactamase extracts were prepared by using sonication (*27*) as described previously (*23*). We performed isoelectric focusing by the method of Matthew et al. (*28*) with an LKB Multiphor apparatus on prepared PAGplate gels (pH 3.5 to 9.5; Amersham Pharmacia Biotech, Hong Kong, China). Enzyme activities of β-lactamases were detected by overlaying the gel with 0.5 mM nitrocefin in 0.1 M phosphate buffer, pH 7.0. We used TEM-1, TEM-10, SHV-1, SHV-5, CMY-1, CTX-M-3, and CMY-2 β-lactamases as standards (*18*,*23*).

### Conjugation Experiments and Plasmid Analysis

Conjugation experiments were performed by the liquid mating-out assay as described (*29*) with streptomycin-resistant *E. coli* C600 as the recipient (*30*). Transconjugants were selected on tryptic soy agar plates supplemented by 500 µg of streptomycin and 10 µg of ceftazidime per milliliter. Plasmids from transconjugants were extracted by using a rapid alkaline lysis procedure (*31*). We analyzed restriction fragment length polymorphism of transferred plasmids using agarose gel electrophoresis of plasmid DNA samples treated with the restriction endonuclease *Eco*RI (Roche Molecular Biochemicals, Mannheim, Germany). The restricted plasmid DNA samples were then transferred to a nylon membrane (Amersham Pharmacia Biotech) and subjected to Southern hybridization. The plasmid sizes of transconjugants were estimated by adding restriction fragments.

### Molecular Techniques

Plasmid preparations from clinical isolates and their transconjugants were used as templates in polymerase chain reaction (PCR) assays. Genes related to *bla*_TEM_, *bla*_SHV_, *bla*_CMY-1_, *bla*_cmy-2_, and *bla*_CTX-M-3_ were amplified with the oligonucleotide primers as described (*23*). Primers 5´-ATAAAATTCTTGAAGACGAAA-3´ and 5´-GACAGTTACCAATGCTTAATCA-3´, corresponding to nucleotides –5 to 15 and 1,074 to 1,053, respectively, of the *bla*_TEM-1_ structural gene (*32*), were used to amplify the entire sequences of *bla*_TEM_-related genes. Primers AmpC-1C (5´-CTGCTGCTGACAGCCTCTTT-3´) and AmpC-1B (5´-TTTTCAAGAATGCGCCAGGC-3´) (*23*), which correspond to nucleotides 28–47 and 1,136–1,117, respectively, of the *bla*_CMY-2_ structural gene (*33*), were used to amplify an internal fragment of approximately 95% of *bla*_CMY-2_–related genes. Both strands of the amplified products were sequenced on an ABI PRISM 310 automated sequencer (Applied Biosystems, Foster City, CA). Colony hybridization and Southern hybridization were performed as described (34,35) with DNA probes prepared from the PCR-generated amplicons. The probes were labeled with [α-^32^P]dCTP (Amersham Pharmacia Biotech) by using the random priming technique with a commercial kit (GibcoBRL Life Technologies, Gaithersburg, MD).

The genetic relatedness of ceftriaxone-resistant *Salmonella* isolates was investigated by ribotyping by using the method described by Popovic et al. (*36*). The chromosomal DNA was extracted and digested overnight with 10 U of *Sph*I and *Pst*I or *Eco*RI (Roche Molecular Biochemicals) (*36*,*37*). A cDNA probe was prepared by reverse transcription of 16S plus 23S rRNA (Roche Molecular Biochemicals) and was labeled with [α-^32^P]dCTP. DNA molecular marker II (Roche Molecular Biochemicals) and 1-kb molecular marker (Promega Corp., Madison, WI) were used as size standards.

## Results

### Emergence of *Salmonella* Isolates Producing CMY-2–Like Enzymes

**Figure 1:**
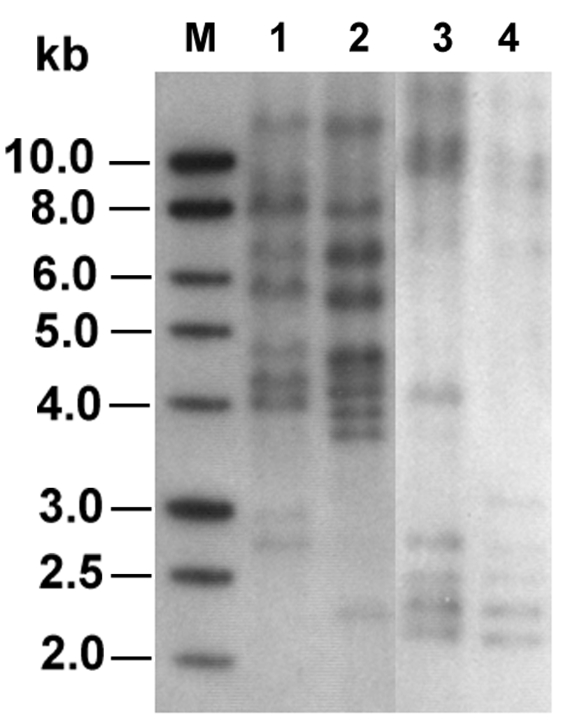
Ribotyping profiles of the two ceftriaxone-resistant *Salmonella* isolates generated by digestion of chromosomal DNA with *Sph*I and *Pst*I (lanes 1 and 2) or *Eco*RI (lanes 3 and 4). Lanes 1 and 3, isolate ST275/00; lanes 2 and 4, isolate ST595/00; lane M, 1-kb DNA ladder (Promega Corp., Madison, WI).

Six of 384 *Salmonella* isolates displayed intermediate resistance to ceftriaxone by the disk diffusion method. The isolates were recovered from stool samples of two patients who had community-acquired enteric infections in August and November 2000, respectively, which were identified as *S. enterica* serovar Typhimurium. One isolate from each patient (isolates ST275/00 and ST595/00) was investigated further. Both isolates demonstrated reduced susceptibilities to ceftazidime (MIC 64 µg/mL), cefotaxime (MIC 16 µg/mL), ceftriaxone (MIC 32 µg/mL), cefoxitin (MIC 128 µg/mL), and amoxicillin-clavulanic acid (MIC 64 µg/mL) and were susceptible to imipenem (MIC 0.25 µg/mL). Both isolates were susceptible to ciprofloxacin and trimethoprim-sulfamethoxazole by the disk diffusion method. Isolate ST275/00 was resistant to chloramphenicol, while isolate ST595/00 was susceptible. Isoelectric focusing showed that both isolates expressed two β-lactamases focusing at pIs 5.4 and 9.0, suggesting that they produced a TEM-1–like enzyme and an AmpC-like β-lactamase. Both isolates yielded an approximately 1.1-kb DNA fragment in PCR with primers for *bla*_CMY-2_–like genes, and the amplified sequences obtained by nucleotide sequencing were identical to the homologous region of *bla*_CMY-2_, which encodes a class C extended-spectrum cephalosporinase (*33*). A narrow-spectrum β-lactamase gene, *bla*_TEM-1_ (*18*) was also detected by PCR and nucleotide sequencing in both isolates. The two ceftriaxone-resistant *Salmonella* isolates had different ribotypes, suggesting that they are of different clones (Figure 1).

### Prevalence of the *bla*_CMY-2_–Like Gene in *E. coli* and *K. pneumoniae* Isolates

**Figure 2:**
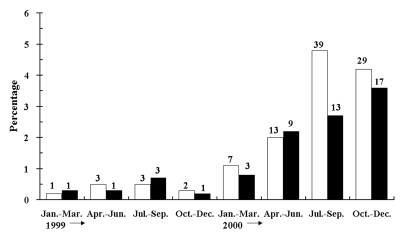
Prevalence rates of the *bla*_CMY-2_–like gene among clinical isolates of *Escherichia coli* (□) and percentage of the new cases infected with or colonized by the *E. coli* isolates producing the CMY-2-like enzyme among patients with *E. coli* isolates (■), 1999 and 2000. Numbers over bars denote the numbers of isolates with a CMY-2–like β-lactamase or the numbers of patients with these isolates.

We found that 659 of 5,520 *E. coli* and 409 of 3,680 *K. pneumoniae* isolates showed resistance to at least one extended-spectrum cephalosporin by the disk diffusion test. These isolates were subjected to colony hybridization and PCR assays. Of these isolates, 97 *E. coli* isolates from 48 patients and 4 *K. pneumoniae* isolates from 2 patients gave a strong signal in colony hybridization with the *bla*_CMY-2_ probe and gave positive results in PCR with the primers for *bla*_CMY-2_. Sequence analysis indicated that the sequences of all amplicons were identical to the homologous region of *bla*_CMY-2_. The prevalence rates of CMY-2–like enzyme producers in *E. coli* increased from 0% in 1997 and 1998 to >4% in late 2000 (Figure 2). Among the patients with *E. coli* isolates, the incidence of patients with *bla*_CMY_-positive isolates increased from 0.0% in 1997 and 1998 to 3.6% in late 2000 (Figure 2).

The sources of *E. coli* isolates harboring a *bla*_CMY-2_–like gene and the infections associated with these isolates are summarized in the Table. The isolates and patients are distributed according to the likely locations of infection or colonization. Of the 97 *bla*_CMY_-positive *E. coli* isolates, 8 isolates were likely acquired in the community by eight patients. Six of these patients had never been hospitalized, and two had been hospitalized 10 months or 2 years before isolation. *K. pneumoniae* isolates producing the CMY-2–like enzyme were associated with nosocomial bloodstream infections in two patients. Notably, *E. coli* (EC811/00) and *K. pneumoniae* (KP218/00) isolates were recovered from the blood sample of a single patient.

### Conjugation Experiments and Plasmid Analysis

One isolate from each patient was subjected to conjugation experiments and plasmid analysis. The *bla*_CMY_-positive plasmids were successfully transferred to *E. coli* C600 from 2 *Salmonella* isolates, 40 of 48 *E. coli* isolates, and 2 *K. pneumonia*e isolates. All *E. coli* transconjugants and their plasmid donors showed decreased susceptibilities to ceftazidime (MIC >32 µg/mL), cefotaxime (MIC >16 µg/mL), ceftriaxone (MIC >32 µg/mL), and cefoxitin (MIC >64 µg/mL). The sizes of the transferred plasmids ranged from approximately 65 kb to >100 kb.

**Figure 3:**
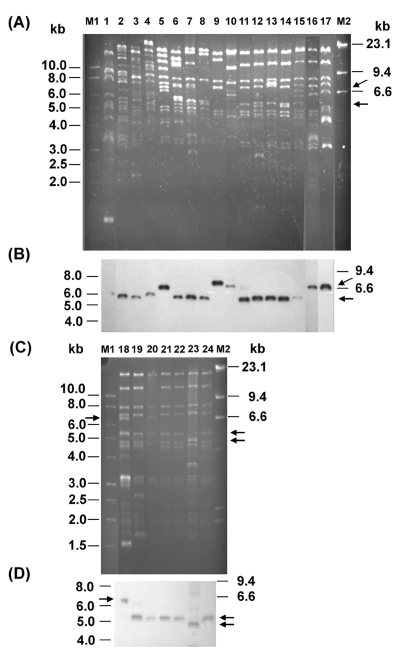
*Eco*RI restriction patterns of plasmids from *Escherichia coli* transconjugants of clinical isolates (A and C) with a CMY-2-like enzyme and *bla*_CMY_ Southern hybridization (B and D). Lanes 1–19, restriction profiles (TP1–TP19) of plasmids from 19 transconjugants of *E. coli* isolates; lanes 20–21, transconjugants of *Salmonella* isolates ST275/00 and ST595/00; lanes 22–23, transconjugants of *K. pneumoniae* isolates KP218/00 and KP1905/00; lane 24, transconjugant of *E. coli* isolate EC811/00; lanes M1 and M2, 1-kb molecular marker and molecular marker II, respectively. Arrows indicate the locations of restriction fragments that were hybridized with the *bla*_CMY_ probe.

Restricted by *Eco*RI, the plasmids from transconjugants of *E. coli* isolates showed 19 restriction patterns, designated TP1–TP19 (Figures 3A and 3C). Patterns TP17 (lane 17) and TP19 (lane 19), the most common patterns, were shown by 6 and 17 transferred plasmids, respectively. Of the 17 isolates with a TP19 plasmid, 3 were acquired from nursing homes and 5 from the community. Of the six isolates with a TP17 plasmid, two could have been acquired from the community. We considered that the remaining isolates with a TP17 or TP19 plasmid were acquired in the university hospital. Each of the remaining 17 patterns was shown by a single transferred plasmid. The TP19 pattern is the only restriction pattern displayed by transconjugants of all three studied bacterial species: *Salmonella* (lane 21), *K. pneumoniae* (lane 22), and *E. coli* (lane 24). Notably, *K. pneumoniae* isolate KP218/00 and *E. coli* isolate EC811/00, from the same patient, both had a TP19-type plasmid. The plasmids from transconjugants of *Salmonella* isolate ST275/00 (lane 20) and another *K. pneumoniae* isolate KP1905/00 (lane 21) showed two distinct restriction patterns. The presence of the *bla*_CMY-2_–like gene on plasmids was confirmed by Southern hybridization with the *bla*_CMY-2_ probe (Figures 3B and 3D).

## Discussion

Emergence of ceftriaxone-resistant salmonellae has become a great public health concern worldwide (*5*–*17*). In Taiwan, no ceftriaxone-resistant isolates were detected from several surveys, which included isolates collected from 1989 to 1998 (*4*,*20*,*21*). One of the surveys was conducted in National Cheng Kung University Hospital (*4*), and one survey conducted in 1998 included isolates from 22 hospitals (*21*). In the present study, *Salmonella* isolates resistant to extended-spectrum β-lactams were not detected until August 2000. Thus, the appearance of ceftriaxone-resistant *Salmonella* strains is likely a recent event.

Production of a CMY-2–like β-lactamase was responsible for resistance to extended-spectrum β-lactams in the *Salmonella* strains we isolated. The spread of *bla*_CMY-2_ in salmonellae recently was reported to be an emerging problem in the United States (*14*,*15*). In Taiwan, the *bla*_CMY-2_–like gene was first detected in *E. coli* isolates collected in 1999 in the university hospital (*23*). The fact that the *bla*_CMY-2_–like gene was found in few isolates from patients with community-acquired infections suggested that the genetic determinant had spread in the community environment (*23*). The appearance of the *bla*_CMY-2_–like gene in the *Salmonella* isolates supports our previous speculation on the spread of the genetic determinant in the community environment. Moreover, the discovery of patients who might have acquired *E. coli* isolates with the CMY-2–like enzyme from nursing homes and in the community (Table) suggests widespread distribution of the *bla*_CMY-2_–like gene in southern Taiwan.

**Table:** Likely sources of *Escherichia coli* isolates producing a CMY-2–like β-lactamase and types of infection associated with the isolates

Specimen or infection	No. (%) of isolates or patients^a^			Total
	NCKU^b^	Nursing home	Community	
Specimen				
Blood	12 (14.4)	2 (33.3)	0 (0)	14 (14.4)
Urine	23 (27.7)	1 (16.7)	6 (75.0)	30 (30.9)
Sputum	17 (20.5)	2 (33.3)	1 (12.5)	20 (20.6)
Wound	23 (27.7)	0 (0)	0 (0)	23 (23.7)
Body fluid	2 (2.4)	1 (16.7)	0 (0)	3 (3.1)
Miscellaneous	6 (7.2)	0 (0)	1 (12.5)	7 (7.2)
Total isolate no.	83 (100)	6 (100)	8 (100)	97 (100)
Infection				
Bacteremia	6 (16.7)	1 (25.0)	0 (0)	7 (14.6)
Urinary tract infection	8 (22.2)	0 (0)	3 (37.5)	11 (22.9)
Pneumonia	1 (2.8)	1 (25.0)	0 (0)	2 (4.2)
Wound infection	9 (25.0)	0 (0)	0 (0)	9 (18.8)
Colonization	12 (33.3)	2 (50.0)	5 (62.5)	19 (39.6)
Total patient no.	36 (100)	4 (100)	8 (100)	48 (100)

^a^Isolates and patients are distributed by the likely locations of infection or colonization.

^b^NCKU, National Cheng Kung University Hospital.

Conjugation experiments and plasmid analysis demonstrated the prevalence of conjugative resistance plasmids TP17 and TP19 among *E. coli* isolates. These *E. coli* isolates were recovered from patients hospitalized in the teaching hospital, transferred from nursing homes, or without recent hospitalization histories (Figure 3). Furthermore, the TP19 plasmid was found in a *Salmonella* strain and a *K. pneumoniae* strain, which suggests the interspecies spread of the *bla*_CMY-2_–like gene among different health-care settings and the community in Taiwan. The dissemination of the resistance determinant is probably partly because of horizontal transfer of endemic resistance plasmids.

All *K. pneumoniae* isolates producing the CMY-2–like enzyme were acquired by patients hospitalized in the university hospital. Producers of the CMY-2–like enzyme in *K. pneumoniae* were not found until 2000 and, even then, remained very rare (*38*). Isolate KP218/00 (lane 22, Figure 3C) and *E. coli* isolate EC811/00 (lane 24, Figure 3C) were obtained from the same patient, and both isolates had a TP19 plasmid, suggesting interspecies spread of a resistance plasmid. Thus, the acquisition of the *bla*_CMY-2_ gene by *K. pneumoniae* in this hospital was likely also a recent event, which occurred after spread of the resistance determinant.

Enterobacteriaceae with plasmid-encoded class C cephalosporinases are typically resistant to cephamycins, extended-spectrum cephalosporins, and monobactams (*19*). No standard methods exist to detect class C cephalosporinases (*39*). Failure to detect and report CMY-2–like enzyme producers, and a lack of infection control measures against such organisms might be partially responsible for the rapid spread of the *bla*_CMY-2_–like gene in *E. coli* and the increased cases of infections caused by such organisms in our hospital (Table). With the discovery of *Salmonella* isolates producing the CMY-2–like enzyme in Taiwan, our study suggests that the cephalosporinase could become an emerging threat, not only to hospitalized patients, but also to public health. Constant and consistent surveillance is needed to prevent its further spread.
